# A novel PSMA-targeting tracer with highly negatively charged linker demonstrates decreased salivary gland uptake in mice compared to [^68^Ga]Ga-PSMA-11

**DOI:** 10.1186/s41181-024-00237-3

**Published:** 2024-01-30

**Authors:** Steve S. Huang, Frank P. DiFilippo, Daniel J. Lindner, Warren D. Heston

**Affiliations:** 1 Cleveland Clinic, 9500 Euclid Ave, Cleveland, OH 44195 USA; 2 Department of Radiology, Mayo Clinic, 13400 E. Shea Blvd., Scottsdale, AZ 85259 USA

**Keywords:** PSMA, GCP-II, Prostate cancer, Salivary gland toxicity

## Abstract

**Background:**

The current generation of radiolabeled PSMA-targeting therapeutic agents is limited by prominent salivary gland binding, which results in dose-limiting xerostomia from radiation exposure. JB-1498 is a urea-based small molecule with a highly negatively charged linker targeting prostate specific membrane antigen (PSMA). Prior work on a similar tracer with the same negatively charged linker demonstrated low normal organ/soft tissue background uptake compared to [^68^Ga]Ga-PSMA-11. The purpose of this study was to investigate if [^68^Ga]Ga-JB-1498 had reduced salivary gland uptake in mice compared to [^68^Ga]Ga-PSMA-11.

**Results:**

JB-1498 demonstrated high affinity for PSMA binding and tumor uptake in a murine tumor model. In an initial biodistribution study with low molar activity, [^68^Ga]Ga-JB-1498 demonstrated salivary gland uptake of 0.13 ± 0.01%ID/g. In a second biodistribution study in non-tumor-bearing mice with high molar activity, [^68^Ga]Ga-JB1498 demonstrated salivary gland uptake of 0.39 ± 0.24% ID/g and kidney activity of 10.12 ± 1.73% ID/g at one hour post IV injection. This salivary gland uptake is significantly less than the published uptake of [^68^Ga]Ga-PSMA-11. Micro-PET visually confirmed the findings of the biodistribution studies. Dynamic micro-PET imaging demonstrated gradually decreasing [^68^Ga]Ga-JB1498 activity in salivary glands and kidneys, compared to gradually increasing [^68^Ga]Ga-PSMA-11 activity in these two organs during the first hour.

**Conclusion:**

Biodistribution and micro-PET imaging of [^68^Ga]Ga-JB-1498 demonstrate significantly decreased salivary gland uptake and different pharmacokinetic behavior in kidneys and salivary glands in mice compared to [^68^Ga]Ga-PSMA-11. Our findings suggest that constructing a PSMA-targeting molecule with a highly negatively charged linker is a promising strategy to reduce salivary gland uptake of GCP-II/PSMA ligands in theranostic applications.

**Supplementary Information:**

The online version contains supplementary material available at 10.1186/s41181-024-00237-3.

## Background

Prostate cancer is the most frequent non-cutaneous cancer and the second most frequent cause of cancer deaths in adult men (Siegel et al. 2023). Metastatic castration-resistant prostate cancer has a poor prognosis, with an estimated 34,700 prostate cancer deaths in the United States in 2023.

Glutamate carboxypeptidase II (GCP-II), or prostate specific membrane antigen (PSMA), is highly expressed in prostate cancers (Bostwick et al. 1998). Positron emission tomography with PSMA-targeting tracers that evolved over the last 20 years has proven to be the most accurate diagnostic modality for detecting metastatic prostate cancer non-invasively (Hope et al. 2019). In addition to PET imaging, therapeutic trials targeting PSMA have been conducted. The landmark VISION trial with [^177^Lu]Lu-PSMA-617 for metastatic castration-resistant prostate cancer demonstrated prolonged survival compared to standard of care (Sartor et al. 2021). The VISION trial led to the FDA approval of [^177^Lu]Lu-PSMA-617 for patients with PSMA-expressing metastatic prostate cancer. Despite the impressive results, the current 7.4 GBq (200 mCi) [^177^Lu]Lu-PSMA-617 regimen is a compromise of efficacy and side effects with 9% complete response and 42% partial response. Replacing ^177^Lu with an alpha emitter (i.e. [^225^Ac]Ac-PSMA-617) can further improve the response (Feuerecker et al. 2021). In fact, one of the first human trials of [^225^Ac]Ac-PSMA-617 demonstrated an impressive response where the PSA value of a patient with metastatic prostate cancer was reduced from 2,923 ng/mL to less than 0.1 ng/mL after 4 cycles of therapy with [^225^Ac]Ac-PSMA-617 (Kratochwil et al. 2016). [^225^Ac]Ac-PSMA-I&T and [^177^Lu]Lu-PSMA-I&T, now emerging from the clinical trial pipeline toward approval, demonstrate similar biodistribution and comparable efficacy/toxicity profile as PSMA-617 based agents (Zacherl et al. 2021; Schuchardt et al. 2022).

A consistent limitation of the current generation of ^225^Ac-PSMA therapeutic agents is the dose limiting xerostomia from salivary gland and lacrimal gland radiation exposure (Kratochwil et al. 2017; Taieb et al. 2018). A GCP-II targeting ligand with reduced salivary gland uptake has the potential to decrease salivary gland toxicity and increase therapeutic window of PSMA targeting radiotherapy.

[^225^Ac]Ac-PSMA-617/[^177^Lu]Lu-PSMA-617 tandem therapy with reduced dose ^225^Ac-PSMA therapeutic agents has been tried in an effort to decrease salivary gland toxicity (Khreish et al. 2020). Many strategies have been employed to try to reduce salivary gland uptake of PSMA-targeting agents. Methods include external cooling (Kalmthout et al. 2018), co-administration of PSMA inhibitors using PMPA (Kratochwil et al. 2015) and unlabeled PSMA-11 (Kalidindi et al. 2021), ductal injection of DCFPyL (Roy et al. 2021), botulinum toxin (Baum et al. 2018), muscarinic inhibition (Mohan et al. 2021), monosodium glutamate administration (Rousseau et al. 2018), and vitamin C administration (Yu et al. 2022). While the above-listed efforts focus on suppressing the salivary gland uptake of PSMA-targeting ligands, there is evidence emerging that salivary gland uptake of PSMA targeting ligands can be greatly reduced by manipulating the molecular framework. Kuo et al. demonstrated that replacing the glutamate sidechain of the Lys-urea-Glu pharmacophore at the catalytic site can lead to significant reduction of salivary gland uptake (Kuo et al. 2022).

Herein, we present preliminary data in mice that constructing a PSMA-targeting molecule with a highly negatively charged linker without altering the glutamate pharmacophore at the catalytic site is another potential strategy to reduce salivary gland uptake.

## Materials and methods

**Fig. 1:**
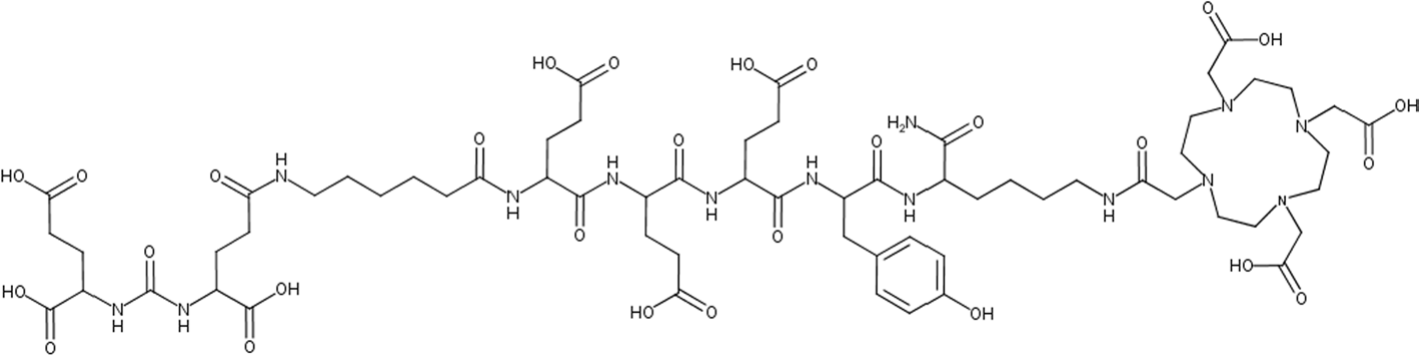
Structure of JB-1498, a PSMA/GCP-II targeting molecule with a highly negatively charged linker between the urea and the DOTA chelate

All chemicals were reagent grade as detailed in the Additional file 1. JB-1498 (Fig. 1) was derived from structure “5” of prior work by Huang et al. (Additional file 1: Fig. 7) by replacing the Bn-NOTA chelate with DOTA (Huang et al. 2014). JB-1498 was synthesized using Fmoc-based solid phase peptide synthesis using Rink amid resin as the solid support and purified by HPLC as detailed in the Additional file 1.

Binding affinity of JB-1498 toward GCP-II was determined using fluorescent enzymatic inhibition assay (Kozikowski et al. 2004) as detailed in the Additional file 1.

In general, radiolabeling can be achieved by heating JB1498 in aqueous sodium acetate solution at pH 4–5 at 95 °C or above for 7–10 min. Radiolabeling reactions were optimized over time. Small variations in conditions in the reactions presented below were results of iterative changes. Please refer to Additional file 1 for HPLC traces of the initial radiolabeling trial (Additional file 1: Fig. 2 and Fig. 3).

Radiolabeling of JB-1498 for the initial biodistribution study was performed using [^68^Ga]GaCl_3_ from a spent ^68^Ge/^68^Ga generator from Isotopen Technologies Garching (Garching, Germany, currently ITM). JB-1498 with [^68^Ga]GaCl_3_ was heated in aqueous sodium acetate buffer at pH 4–5 and 97  C for 7 min. The reaction product was HPLC-purified for the biodistribution study as detailed in the Additional file 1. The molar activity of the purified product was less than 7.4 GBq/μmol (200 Ci/mmol) at the conclusion of synthesis.

For the high molar activity biodistribution and imaging study, a fresh 1.85 GBq (50mCi) GalliaPharm Gallium-68 generator from Eckert & Ziegler (Berlin, Germany) was used. Radiolabeling of JB-1498 with [^68^Ga]GaCl_3_ was performed in aqueous sodium acetate buffer at pH 4–5 and 98 °C for 10 min. The reaction product was diluted with water, loaded onto an Oasis HLB cartridge, washed with water, and eluted with 2:1 PBS:EtOH as detailed in the Additional file 1. The fractions with highest radioactivity were diluted with saline for injection in mice. Please refer to Additional file 1: Figs. 4 and Fig. 5 for HPLC traces of the products prior to and after solid phase extraction. The molar activity of the purified product was estimated to be 59 GBq/μmol (1600 Ci/mmol) at the time of first injection. [^68^Ga]Ga-PSMA-11 was produced concurrently by incubating [^68^Ga]GaCl_3_ and PSMA-11 precursor in sodium acetate buffer at pH 4–5 at room temperature for 10 min. Please refer to Additional file 1: Fig. 6 for HPLC trace of the product. The molar activity of the product was estimated to be 48 GBq/μmol (1300 Ci/mmol) at the time of first injection.

For the initial biodistribution study in tumor bearing mice, 3 male 10 week-old NSG mice (NOD.Cg-*Prkdc*^*scid*^*Il2rg*^*tm1Wjl*^/SzJ) were inoculated subcutaneously with 4 million cells of PC3-PIP PSMA-expressing cell line. Mice were housed in a specific pathogen free, temperature- and humidity-controlled environment, in standard micro-isolator top cages with ad libitum access to chow. Biodistribution studies were conducted when the long axis of the tumor exceeded 1 cm. Each mouse was injected with 100 μL of the [^68^Ga]Ga-JB-1498 solution under isoflurane anesthesia via retro-orbital injection in the right eye and euthanized 1 h after injection. Major organs (including the left salivary glands) were dissected for radioactivity quantification. The dose injected was 0.74 ± 0.27 MBq.

For the high molar activity biodistribution study aimed at maximizing salivary gland uptake, 3 male NSG mice without tumor were injected with 100μL of the [^68^Ga]Ga-JB-1498 solution under anesthesia via retro-orbital injection per the above-described protocol. The dose injected was 1.31 ± 0.26 MBq.

For the micro-PET imaging study, high molar activity [^68^Ga]Ga-JB-1498 and [^68^Ga]Ga-PSMA-11 were administered to non-tumor bearing NSG mice via tail vein injection. The dose injected was 5.3MBq of [^68^Ga]Ga-PSMA-11 and 4.4 MBq of [^68^Ga]Ga-JB-1498. Imaging was performed on a nanoScan PET/CT instrument (Mediso USA, Arlington, Virginia). List mode PET acquisition was performed for 45 min starting 25 min post-injection. CT was performed for PET attenuation correction and anatomic localization.

## Results

By mass spectrometry, the singly charged form of the purified JB-1498 sample had a mass of 1497.66 Da, matching the expected molecular structure of JB-1498 (Fig. 1). Using fluorescent enzymatic assay, EC_50_ of JB-1498 was estimated to be 287 ± 64 pM (Additional file 1: Fig. 1). This places the upper limit of K_*i*_ of JB1498 to PSMA = 300 pM (K_*i*_ is lower than 300 pM).

The initial biodistribution study performed using an aged ITG Ga-68 generator (with estimated molar activity less than 7.4 GBq/μmol or 200 Ci/mmol) demonstrated tumor uptake of 12.09 ± 1.28%, salivary gland uptake of 0.13 ± 0.01% and liver uptake of 0.20 ± 0.09% (Additional file 1: Table 1). We conducted an additional biodistribution study in non-tumor-bearing-mice with high molar activity [^68^Ga]Ga-JB1498 at one hour post IV tracer injection and demonstrated salivary gland activity of 0.39 ± 0.24% ID/g and kidney activity of 10.12 ± 1.73% ID/g (Additional file 1: Table 2). The uptake values in these two organs are much lower than the [^68^Ga]Ga-PSMA-11 salivary gland uptake of 10.00 ± 2.52% ID/g and kidney uptake of 182.0 ± 33.5% ID/g at one hour after injection as published by Rousseau et al. (2018) (Additional file 1: Table 3). Please note that the strain of mouse in this study is identical to that used by Rousseau et al. The molar activity and injected doses are all within the range specified by Rousseau et al.

**Fig. 2:**
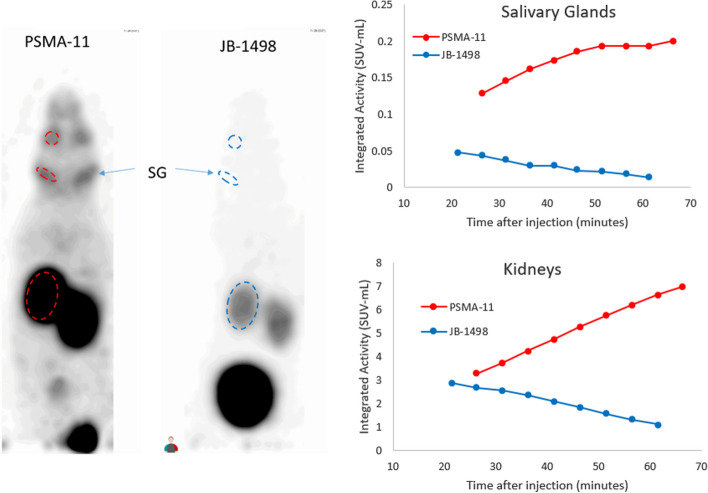
Left panel: [^68^Ga]Ga-PSMA-11 and [^68^Ga]Ga-JB-1498 head-to-head comparison micro-PET images at 1 h post injection, with same SUV grayscale. There is very little salivary gland (SG) and background activity in the mouse injected with [^68^Ga]Ga-JB-1498 compared to [^68^Ga]Ga-PSMA-11. Most of the injected [^68^Ga]Ga-JB-1498 is in the urinary bladder due to rapid tracer excretion via the kidneys. Right Panel: representative time-activity curve of [^68^Ga]Ga-PSMA-11 and [^68^Ga]Ga-JB-1498 in salivary glands (combined parotid and submandibular glands) and kidneys during the first hour of micro-PET imaging. The diverging time activity curve reflects active salivary gland and renal cortical uptake of [^68^Ga]Ga-PSMA-11 and lack of specific uptake of [^68^Ga]Ga-JB-1498 in these two organs

Micro-PET images of mice at 1 h post-injection of [^68^Ga]Ga-JB-1498 and [^68^Ga]Ga-PSMA-11 visually confirmed much lower salivary gland and kidney uptake with [^68^Ga]Ga-JB-1498 compared to [^68^Ga]Ga-PSMA-11 (Fig. 2).

## Discussion

The need to reduce salivary gland toxicity associated with current GCP-II catalytic site targeted treatment is widely recognized. However, the ability to systematically test a GCP-II targeting compound’s propensity for salivary gland uptake was not available when the issue was first recognized. Rousseau et al. first demonstrated a testable experimental system where salivary gland uptake was consistently reproduced in mice with [^68^Ga]Ga-PSMA-11 at 82.6 ± 44.3 GBq/μmol (2230 ± 1200 Ci/mmol) molar activity (Rousseau et al. 2018). Roy et al. validated mice as a testable experimental model for salivary gland uptake using ^18^F-DCFPyL (molar activity of 44–96 GBq/μmol or 1200–2600 Ci/mmol) (Roy et al. 2020).

We had designed GCP-II inhibitors with highly negatively charged linkers with three glutamates a decade ago (Huang et al. 2014; Laydner et al. 2013). A ^18^F-AlF labeled variant of the negatively charged linkers demonstrated rapid renal parenchymal washout and generally low normal organ activity at one hour post injection compared to other tracers published at the time. Initial PET imaging at the time demonstrated no significant salivary gland uptake. We were intrigued by the findings and subsequently constructed a variant of the molecule by replacing the Bn-NOTA chelate with DOTA (JB-1498). Replacement of the thiourea with the amide bond for chelator conjugation was necessary due to the propensity for radiolysis of the radiolabeled compound at high molar activity (see Additional file 1: Figs. 8–11).

In the initial biodistribution experiment, [^68^Ga]Ga-JB-1498 demonstrated near background activity in salivary glands in mice and much lower renal cortical activity compared to published biodistribution data for [^68^Ga]Ga-PSMA-11 (Rousseau et al. 2018). This correlates well with the imaging and biodistribution study of the ^18^F-AlF labeled variant of the molecule with a negatively charged linker (Huang et al. 2014). However, our initial biodistribution studies were not in the range of molar activity reported in literature where [^68^Ga]Ga-PSMA-11 demonstrated significant salivary gland uptake. Molar activity of PSMA-targeting ligands has been shown to affect organ uptake in mouse biodistribution studies (Khreish et al. 2020; Wurzer et al. 2018). To allow for maximal salivary gland uptake of ^68^Ga-JB-1498 in high molar activity, we conducted a biodistribution study in non-tumor bearing mice using the eluate of a new ^68^Ga generator for radiolabeling of JB-1498. A fresh ^68^Ga generator allowed us to produce high molar activity [^68^Ga]Ga-JB-1498 specified by Rousseau et al. (2018), Roy et al. (2020). We used non-tumor-bearing mice for the high-specific-activity experiment to maximize normal organ uptake. Salivary gland uptake of [^68^Ga]Ga-JB1498 at high molar activity increased slightly compared to the lower molar activity experiment, indicating there is some effect of competition by the cold compound in mice. Nevertheless, [^68^Ga]Ga-JB-1498 exhibited much lower salivary gland and renal cortical uptake compared to [^68^Ga]Ga-PSMA-11. Dynamic micro-PET imaging demonstrates drastic differences in pharmacodynamic behavior in the salivary gland and renal cortex between [^68^Ga]Ga-JB-1498 and [^68^Ga]Ga-PSMA-11.

The cause of salivary gland uptake has been debated in the literature (Lucaroni et al. 2023; Lee et al. 2023; Huang et al. 2023; Bassi et al. 2023). The mechanisms of salivary gland uptake and, for that matter, the mechanisms of reduction of salivary gland uptake are still unclear. At first glance, our work seems to suggest that salivary gland uptake of PSMA-11 is unrelated to PSMA. However, one should be cautious that findings in mice may not translate to humans and further studies are needed. Our findings indicate that there may be several methods of structural modification targeting different regions that have the potential to alter salivary gland and renal cortex tracer uptake of PSMA-targeting small molecules. One is the replacement of the glutamate sidechain of the Lys-urea-Glu pharmacophore demonstrated by Kuo et al. (2022). Of note, the glutamate pharmacophore altered by Kuo et al. has been “conserved” in all urea-based high affinity PSMA targeting agents since the publication of its synthetic method by Kozikowski et al. (2001). Optimization of halogen positioning within the molecular framework of the inhibitor without altering the glutamate pharmacophore has also led to reduction of salivary gland uptake in mice (Mease et al. 2022). A potential new target of structural modification without altering the glutamate pharmacophore is the overall charge in the linker region as illustrated herein.

There is much yet to learn about tracer interaction with GCP-II/PSMA or possibly other GCP-like molecules. We have high hopes that future studies will lead to significant reduction of salivary/lacrimal gland toxicity, and profoundly widen the therapeutic window of PSMA-targeted therapies.

### Supplementary Information

**Additional file 1**. Supplemental Data.

## Data Availability

Detailed methods, binding and biodistribution data are included in the Additional file 1. Additional data will be made available on reasonable request.
