# Supplementary material for: A novel PSMA-targeting tracer with highly negatively charged linker demonstrates decreased salivary gland uptake in mice compared to [68Ga]Ga-PSMA-11
Source: EJNMMI Radiopharm Chem. 2024 Jan 30;9:7. doi: 10.1186/s41181-024-00237-3 (PMC10828251; doi:10.1186/s41181-024-00237-3)
Supplement: Supplementary file 1 — Additional file 1. Supplemental Data. [file 41181_2024_237_MOESM1_ESM.docx]

Supplemental data

**Materials:**

Fmoc-L-Lys-mono-amid-DOTA-tris(t-Bu ester) was purchased from Macrocyclics (Plano, TX, USA), Fmoc-Tyr(tBu)-OH and H-Glu(OtBu)-OtBu were purchased from Bachem (Torrance, CA, USA), Fmoc-D-Glu(OtBu)-OH and Fmoc-ε-Aca-OH and Rink Amide MBHA resin was purchased from Peptides International (Louisville, KY, USA), HCTU (O-(1H-6-Chlorobenzotriazole-1-yl)-1,1,3,3-tetramethyluronium hexafluorophosphate) was purchased from Novbiochem (now part of MilliporeSigma, Burlington, MA, USA). DMF (N, N- Dimethylformamide, HPLC grade) was purchased from Alfa Aesar (now part of Thermo Scientific Chemicals, Waltham, MA, USA). DIPEA (N,N-Diisopropylethylamine), piperidine, TIS (Triisopropylsilane), Diethyl Ether were purchased from Sigma-Aldrich (St. Louis, MO, USA), Trifluoroacetic Acid, glacial acetic acid and Ultrapur water were purchased from EMD Millipore (now part of MilliporeSigma, Burlington, MA, USA), Triphosgene was purchased from Chem-Impex International (Wood Dale, IL, USA). Ethylene chloride (dichloromethane, DCM) was purchased from Fisher Chemical (Waltham, MA, USA). Acetonitrile was purchased from J.T. Baker (part of VWR, Radnor, PA, USA). PSMA-11 precursor was purchased from ABX Advanced Biochemical Compounds Biochemische Forschungsreagenzien GmbH (Radeberg, Germany). All chemicals were of reagent grade quality or higher.

**Synthesis of JB-1498**

JB1498 was synthesized by first performing seven cycles of Fmoc-peptide synthesis using a Rink amide resin followed by on-resin urea formation. A 0.1 mmol scale synthesis was performed manually. At the beginning of each coupling cycle, the Fmoc protecting group was removed with 20% piperidine in DMF. For the first cycle, Fmoc-L-Lys-mono-amid-DOTA-tris(t-Bu ester) (100mg, Macrocyclics) was reacted with 0.4 gram of rink amide resin (0.45 mmol/g or 0.18 meq) in the presence of HCTU [(O-(1H-6-Chlorobenzotriazole-1-yl)-1,1,3,3-tetramethyluronium hexafluorophosphate]. The first cycle was followed by capping of unreacted primary amine on the Rink amide resin using acetic acid, HCTU and DIPEA. After the capping cycle, the synthesis proceeded with standard Fmoc peptide synthesis cycles with the coupling of Fmoc- Tyr(tBu)-OH, Fmoc-D-Glu (OtBu)-OH, Fmoc-D-Glu (OtBu)-OH, Fmoc-D-Glu (OtBu)-OH, Fmoc-Ahx-OH, Fmoc-Glu-OtBu. After Fmoc removal of product from the seventh cycle with 20% piperidine in DMF, the resin was washed with DMF and DCM and remained soaked in DCM until ready for reaction with isocyanate. For isocyanate formation, two vials were prepared on dry-ice: Vial A: 0.046 gram of Triphosgene in 1mL DCM and Vial B: 0.151 gram of H-Glu(OtBu)-OtBu in 1mL DCM + 223 μL of DIPEA. Solution B was added to A dropwise on dry ice. For on-resin urea formation, the isocyanate solution was warmed to room temperature. After 30 minutes at room temperature, 112 μL DIPEA was added to the isocyanate solution. The isocyanate solution was poured onto the resin and incubated at room temperature for 1 hour for urea formation. The resin was then washed with DCM, DMF and Methanol. For cleavage and de-protection, 5.0mL of cleavage mixture using TFA/TIS/H2O (95% / 2.5% / 2.5%) was reacted with resin for 4 hours at room temperature. The cleavage mixture was collected and TFA was evaporated with airstream. The residual liquid was precipitated with diethyl ether. The solid crude product was collected, air dried and further dried under vacuum.

High performance liquid chromatography (HPLC) was performed on a Shimadzu HPLC system equipped with a SPD-20V Prominence UV/visible detector and monitored at a wavelength of 260 nm (Shimadzu Scientific Instruments, Columbia, MD). Semi-Preparative HPLC was achieved using an analytical Symmetry C18 column (150mm 4.6mmx5mm, Waters Corporation, Milford, MA) with 1.5 ml/min flow rate and a gradient of 10%B-14%B (where solvent B is acetonitrile and solvent A is water with 0.1% formic acid). Analytical HPLC was performed using an analytical Phenomenex Synergi Hydro-RP 80A 150x4.6mm 4 micrometer column (Phenomenex, Torrance, CA).

Mass spectrometry was performed by the Lerner Research Institute Proteomics Laboratory of the Cleveland Clinic Foundation.

**Competitive Binding Assay**

PC3-PIP PSMA expressing cells were grown to confluency, harvested in 50mM Tris-HCl, pH 7.5 and subjected to 4 freezing and thawing cycles. Cell membranes were washed three times by centrifugation at 12,000g or higher. The cell membrane mixture was diluted to a concentration of 50 mg/mL in 50mM Tris-HCl, pH 7.5 (based on wet weight of the solid after centrifugation) and stored in a -80°C freezer until ready to use.

The assays were conducted on 96-well plates. In the first phase of the assay, each well contained 100 μL 50mM Tris-HCl, pH 7.5 with 4uM NAAG (N-Acetylaspartylglutamate, Millipore-Sigma, Burlington, MA) and 10 μg of PC3-PIP membrane and different concentrations of JB-1498. The 96-well plate was incubated at 37 °C for 2 hours. Then the glutamic acid concentration was measured with Amplex Red glutamic acid assay kit (Invitrogen-Molecular Probes, now part of Thermo Fisher Scientific, Waltham, MA)) using a fluorescent plate reader (excitation at 530nm, measuring emission at 590nm). The fluorescence signals at 590nm were then normalized and normalized fluorescence was plotted against the Log concentration of JB1498 ligand (Figure 1). EC_50_ was determined using the equation: Fluorescence = baseline + (max fluorescence – baseline) *[1+1/(EC50/Ligand concentration)]. Microsoft Excel and KaleidaGraph (Synergy Software, Reading, PA) were used for data processing and graphing.


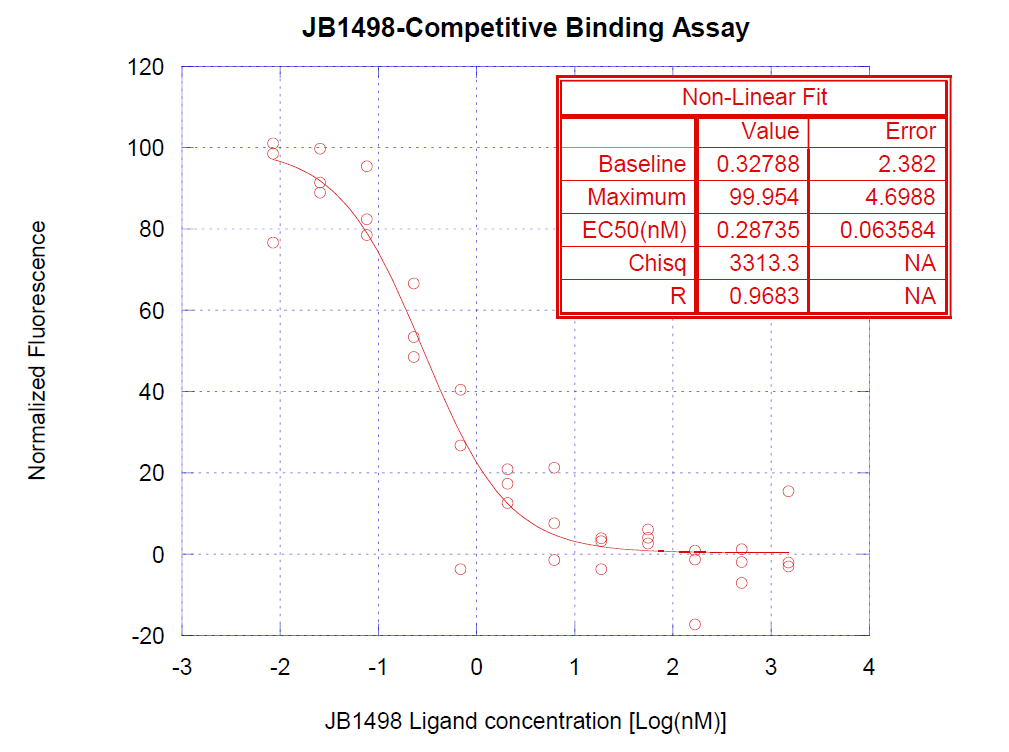


Figure 1. JB1498 competitive binding assay. Normalized fluorescence was plotted against the Log concentration of JB1498 ligand. EC_50_ was determined to be 287±63 pM.

**Radiolabeling:**

In general, radiolabeling can be achieved by heating JB1498 in aqueous sodium acetate solution at pH 4-5 at 95°C or above for 7-10 minutes. Radiolabeling reactions were optimized over time. Small variations in conditions in the reactions presented below were results of iterative changes. Experimental conditions are presented as recorded.

In an early [^68^Ga]Ga-JB-1498 radiolabeling trial. A vial containing 2.5 μL of JB1498 (0.17mg/mL), 5 μL of NaOAc 1M aqueous solution, 5 μL of NaOH 1M aqueous solution, and 0.2 mL of ITG Ga-68 generator eluate was heated at 95°C for 10 minutes. HPLC was performed with a flow rate of 1.0 ml/min, gradient of 5%-35%B in 15 minutes where solvent B is acetonitrile and solvent A is water with 0.1% formic acid. Column: Waters (Milford, MA) Symmetry C18, 5μm, 4.6 x 150 mm column. A single radioactive peak was observed at 10.5 minutes (Figure 2). The identity of the peak was verified with ^nat^Ga-JB-1498 synthesized in microscale by mixing 10 μL of JB1498 (2.4mg/mL) with 3 μL of Ga(NO_3_)_3_ 2.5mg/mL and heating the aqueous solution at 97°C for 30 minutes. The heated mixture was then diluted with 95 μL water and injected for HPLC analysis under identical gradient conditions as described above. The UV peak of ^nat^Ga-JB-1498 at 10.5 min corresponds to the radioactive peak [^68^Ga]Ga-JB-1498 under identical HPLC conditions (Figure 3). LCMS indicated the 10.5-minute peak had MW of 1565 (z+1), which is expected for ^nat^Ga-JB-1498.

Figure 2. Initial [^68^Ga]Ga-JB-1498 radiolabeling demonstrating a single radioactive peak at 10.5 minutes. HPLC was performed with flow rate of 1.0 ml/min, gradient of 5%-35%B in 15 minutes where solvent B is acetonitrile and solvent A is water with 0.1% formic acid. Column: Waters (Milford, MA) Symmetry C18, 5μm, 4.6 x 150 mm column.

Figure 3. Microscale synthesis of ^nat^Ga-JB-1498 demonstrating a single dominant UV peak at 10.5 minutes. Undulation of UV signals near solvent front is related to presence of Ga(NO_3_)_3_. LCMS demonstrates the 10.5-minute peak has MW of 1565 (z+1), which is expected for ^nat^Ga-JB-1498.

Radiolabeling of JB-1498 biodistribution study (low molar activity): Labeling reaction vial containing 2.5 μL of JB1498 (2.4mg/mL), 250 μL of NaOAc buffer B (a solution consisting of 1000 μL of 1M NaOH and 60 μL of glacial acetic acid) and 1.0mL of ITG Ga-68 generator eluate (approximately 37 MBq/mL or 1mCi/1mL) was heated at 97°C for 7 minutes. The aqueous solution was concentrated by rotary evaporator with gentle heating for 10 minutes. The mixture was loaded onto HPLC and purified with analytical HPLC with gradient 9%B-35%B in 15 minutes with flow rate of 1.5ml/min (where solvent B is acetonitrile and solvent A is water with 0.1% formic acid). Peak at 6.3 minutes was collected and acetonitrile was evaporated with rotary evaporator. Residual formic acid in the 0.35 mL solution was buffered with 5 μL of 1M NaOAc and 6 μL of 1M NaOH and diluted 1:1 with phosphate buffered saline (PBS). Final pH was 4-5 by pH paper. The estimated molar activity is below 7.4 GBq/μmol or 200 Ci/mmole.

Please note that the differences of radiolabeling and purification methods between the low-molar-activity preparation described above and the high-molar-activity preparation described below is due to evolving radiolabeling routines as we transitioned from one generator to another. HPLC purification of Ga-68 JB1498 was an early attempt to boost specific activity of the product; this strategy did not work. The strategy that ultimately worked for generating high molar activity batches was to use a fresh GalliaPharm generator, which allows maximal radioactivity with minimal precursor as described below. A simple solid phase extraction method was used to minimize residual [^68^Ga]GaCl_3_ as described below.

Radiolabeling of JB-1498 (high molar activity): The labeling reaction contained 8 μL of JB1498 (0.2mg/mL), 160 μL of NaOAc Buffer B and 0.8mL of Eckert & Ziegler GalliaPharm Generator eluate (approximately 4mCi/1mL). NaOAc Buffer B is a solution consisting of 1000 μL of 1M NaOH and 60 μL of glacial acetic acid) with a final pH of 4-5 by pH paper. The mixture was heated at 98°C for 10 minutes. HPLC analysis of the product demonstrated 82% labeling efficiency (Figure 4) with 18% of activity at the solvent front indicating the presence of unbound [^68^Ga]GaCl_3_. The aqueous solution was diluted with water and loaded on to HLB column (Waters, Milford, MA) and washed with 5mL of water followed by elution with 2:1 phosphate buffered saline (PBS): ethanol in 0.2-0.3 mL fractions. The post SPE product demonstrated no visible radioactive peak at the solvent front (Figure 5). The product was diluted with normal saline before injection for biodistribution study. Specific activity was 59 GBq/μmol or 1600 Ci/mmol at the time of first animal injection. Specific activity was calculated based on the total amount of JB-1498 used (1.6 μg, 1.07 x 10^-9^M), radioactivity decay-corrected to the time of first injection 78 MBq (2.1 mCi) and a multiplication factor of 0.82 to account for 82% efficiency of radiolabeling.

Figure 4. Radio-HPLC trace of ^68^Ga-JB-1498 after high molar activity radiolabeling prior to SPE, demonstrating approximately 82% efficiency with expected product peak at 5.7 minutes and approximately 18% radioactivity near solvent front consistent with unbound [^68^Ga]GaCl_3_. The radiochemical purity is likely underestimated since the major peak height signal appears to be saturated at 1.4 x10^6^ μV; the minor radioactive products are likely exaggerated. Injection was not repeated due to time constraints.

Figure 5 Radio-HPLC trace of [^68^Ga]Ga-JB-1498 after high molar activity radiolabeling after Solid phase extraction. Unbound [^68^Ga]GaCl_3_ seen in Figure 4 was filtered out with solid phase extraction with HLB column (Waters, Milford, MA). Analytical HPLC was performed with flow rate of 1.5 ml/min, gradient of 5%-95%B in 15 minutes where solvent B is acetonitrile and solvent A is water with 0.1% formic acid. Column: Phenomenex Synergi Hydro-RP 80A 150x4.6mm 4 micrometer column (Phenomenex, Torrance, CA).

Radiolabeling of PSMA-11 (high specific activity): The labeling reaction contained 3 μL of PSMA-11 (0.5mg/ML, ABX), 160 μL of NaOAc buffer B and 0.8mL of Eckert & Ziegler GalliaPharm Generator eluate. The final pH was 4-5 by pH paper. The mixture was left at room temperature for 10 minutes. HPLC analysis demonstrated near 100% efficiency with no visible radioactivity near the solvent front (Figure 6). The product was diluted with normal saline before injection for biodistribution study.

Figure 6. Radio-HPLC trace of [^68^Ga]Ga-PSMA 11 after radiolabeling, demonstrating near 100% efficiency with no visible free [^68^Ga]GaCl_3_. Analytical HPLC was performed with flow rate of 1.5 ml/min, gradient of 5%-95%B in 15 minutes where solvent B is acetonitrile and solvent A is water with 0.1% formic acid. Column: Phenomenex Synergi Hydro-RP 80A 150x4.6mm 4 micrometer column (Phenomenex, Torrance, CA).

Table 1 Biodistribution of [^68^Ga]Ga-JB-1498 in PSMA+ tumor-bearing NSG mice 1 hour after IV injection of [^68^Ga]Ga-JB-1498 (specific activity below 7.4 GBq/μmol or 200 Ci/mmole).

Unit %ID/gram of tissue.

| Blood |  | 0.22% | ± | 0.02% |
| --- | --- | --- | --- | --- |
| Muscle |  | 0.22% | ± | 0.23% |
| Bone |  | 0.11% | ± | 0.04% |
| Liver |  | 0.20% | ± | 0.09% |
| Kidney* |  | 12.98% |  |  |
| Spleen** |  | 0.31% | ± | 0.12% |
| Heart |  | 0.12% | ± | 0.01% |
| Lung |  | 0.24% | ± | 0.05% |
| Salivary gland | | 0.13% | ± | 0.01% |
| Tumor |  | 12.09% | ± | 1.28% |

* Single sample of left kidney mistaken for spleen. We originally planned not to disturb the urinary system given known intense activity in the urinary system which has the potential to contaminate other samples.

** 2 splenic samples instead of 3 given the mistake noted above. The activity had decayed by the time the data were analyzed and mistake was realized. The third spleen was not retrieved.

Table 2, Biodistribution of [^68^Ga]Ga-JB-1498 in non-tumor-bearing NSG mice 1 hour after IV injection of high specific activity [^68^Ga]Ga-JB-1498 (N=3). (Specific activity was 59 GBq/μmol or 1600 Ci/mmol at the time of first animal injection).

Unit: %ID/gram of tissue.

| Organ |  |  |  |  |
| --- | --- | --- | --- | --- |
| Blood |  | 0.39% | ± | 0.06% |
| parotid (Left) |  | 0.36% | ± | 0.11% |
| submandibular (Left) |  | 0.42% | ± | 0.31% |
| Liver |  | 0.68% | ± | 0.13% |
| Kidney |  | 10.12% | ± | 1.73% |
| Spleen |  | 2.34% | ± | 0.46% |
| Lung |  | 0.66% | ± | 0.10% |
| Combined Salivary gland |  | 0.39% | ± | 0.24% |

Table 3. Comparing [^68^Ga]Ga-JB-1498 with [^68^Ga]Ga-PSMA-11 in NSG mice Unit: %ID/gram of tissue.

|  |  | [^68^Ga]Ga-JB-1498 (Low specific activity) | | |  |  | [^68^Ga]Ga-JB-1498 (high specific activity) | | |  |  | [^68^Ga]Ga-PSMA 11 *(from Rousseau et. Al) | | |
| --- | --- | --- | --- | --- | --- | --- | --- | --- | --- | --- | --- | --- | --- | --- |
| Blood |  | 0.22% | ± | 0.04% |  |  | 0.39% | ± | 0.06% |  |  | 0.31% | ± | 0.08% |
| Muscle |  | 0.19% | ± | 0.20% |  |  |  |  |  |  |  | 0.54% | ± | 0.14% |
| Bone |  | 0.10% | ± | 0.04% |  |  |  |  |  |  |  |  |  |  |
| Liver |  | 0.18% | ± | 0.08% |  |  | 0.68% | ± | 0.13% |  |  | 0.44% | ± | 0.13% |
| Kidney |  | 12.98% |  |  |  |  | 10.12% | ± | 1.73% |  |  | 182.00% | ± | 33.50% |
| Spleen |  | 0.27% | ± | 0.10% |  |  | 2.34% | ± | 0.46% |  |  | 22.20% | ± | 7.91% |
| Heart |  | 0.11% | ± | 0.01% |  |  |  |  |  |  |  |  |  |  |
| Lung |  | 0.21% | ± | 0.05% |  |  | 0.66% | ± | 0.10% |  |  |  |  |  |
| Salivary gland | | 0.11% | ± | 0.01% |  |  | 0.39% | ± | 0.24% |  |  | 10.00% | ± | 2.52% |
| Tumor |  | 10.49% | ± | 1.11% |  |  |  |  |  |  |  | 8.67% | ± | 1.97% |

* Rousseau E, Lau J, Kuo HT, Zhang Z, Merkens H, Hundal-Jabal N, et al. Monosodium Glutamate Reduces (68)Ga-PSMA-11 Uptake in Salivary Glands and Kidneys in a Preclinical Prostate Cancer Model. J Nucl Med. 2018;59:1865-8. doi:10.2967/jnumed.118.215350. Note that Rousseau et al. also used NSG mice (NOD.Cg-*Prkdc^scid^Il2rg^tm1Wjl^*/SzJ). The difference between the mouse models is that Rousseau et al. used the LNCaP cell line while PC3-PIP was used in this work.

Assessment of stability.

The compound from which JB-1498 was developed (Figure 7) demonstrated multiple peaks in radio-HPLC when labeled in aqueous sodium acetate buffer without radioprotectant. The compound demonstrated reasonable stability with minimal degradation with the addition of ascorbic acid as a radioprotectant (Figure 8 and 9). [^68^Ga]Ga-JB-1498 demonstrated no significant degradation in aqueous sodium acetate buffer without radioprotectant (Figure 10 and 11). The compound in Figure 7 was not further developed as a potential theranostic agent since the need for a radioprotectant with Ga-68 labeling indicated potentially significant instability with high dose beta and alpha emitters.


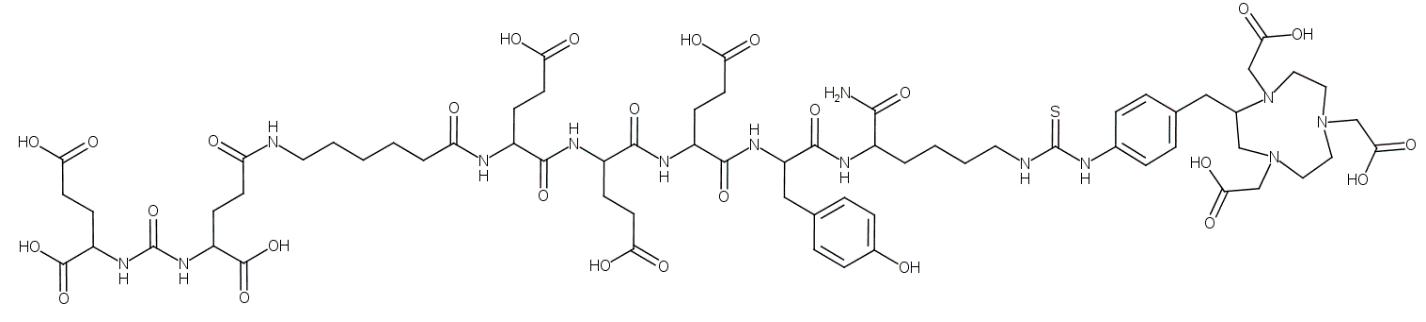


Figure 7. The compound from which JB-1498 was developed. This is structure “5” of Huang et al, 2014 (PMID: 24615708 DOI: 10.1002/pros.22789).


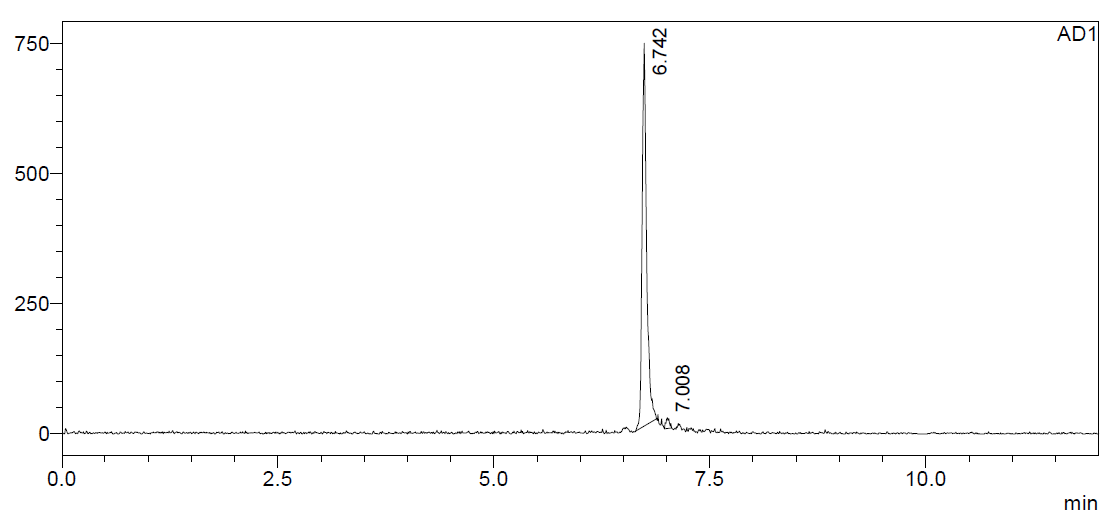


Figure 8. Radio-HPLC trace of the compound in Figure 7 seventy minutes after labeling with Ga-68. The labeling reaction consisted of 2 μL of the compound (0.5mg/mL), 40 μL of NaOAc Buffer B, 5 μL ascorbic acid (100mg/mL) and 0.2mL of Eckert & Ziegler GalliaPharm Generator eluate. Buffer B composition is stated above. The HPLC gradient used was 5%-95%B in 15 minutes where solvent B is acetonitrile and solvent A is water with 0.1% formic acid. Column: Phenomenex Synergi Hydro-RP 80A 150x4.6mm 4 micrometer column (Phenomenex, Torrance, CA).


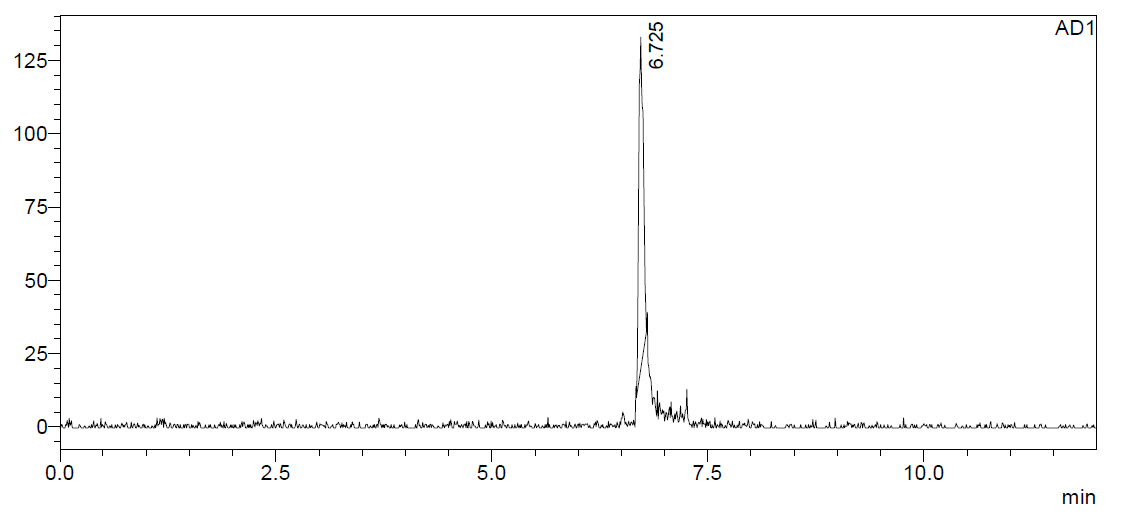


Figure 9. Radio-HPLC trace of the compound in Figure 7 two hours after radiolabeling in the presence of ascorbic acid, demonstrating some radiolysis even in the presence of a radioprotectant.


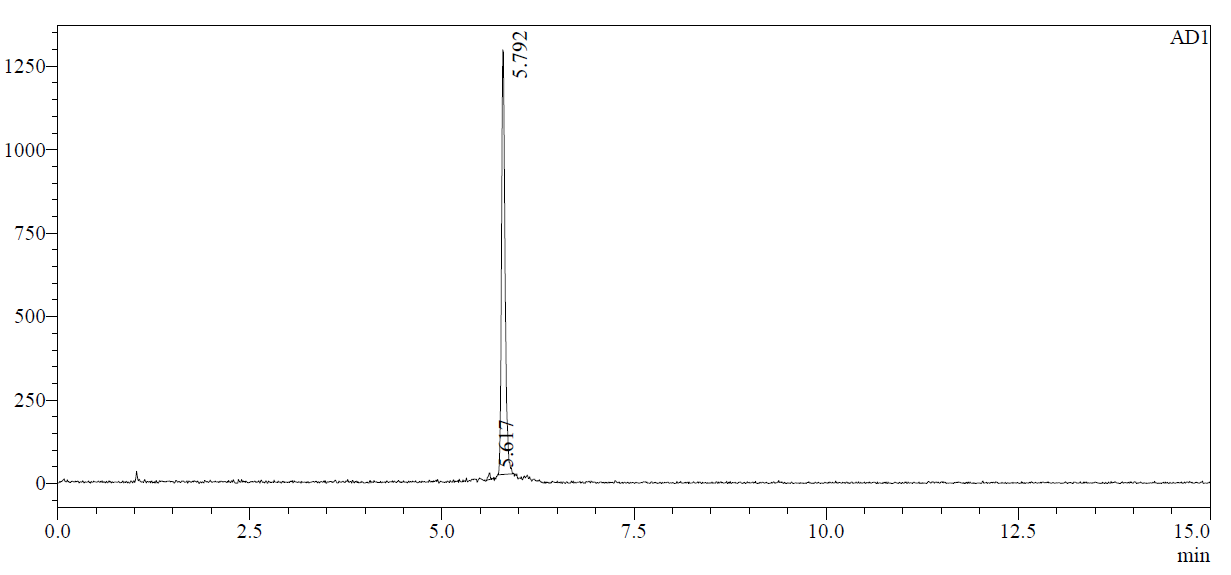


Figure 10. Radio-HPLC trace of [^68^Ga]Ga-JB-1498 immediately after radiolabeling. The small signal at the solvent front is a very small amount of [^68^Ga]GaCl_3_. The labeling reaction contained 2 μL of JB1498 (0.5mg/mL), 40 μL of NaOAc Buffer B and 0.2mL of Eckert & Ziegler GalliaPharm Generator eluate. Buffer B composition is stated above. The HPLC gradient used was 5%-95%B in 15 minutes where solvent B is acetonitrile and solvent A is water with 0.1% formic acid. Column: Waters (Milford, MA) Symmetry C18, 5μm, 4.6 x 150 mm column.


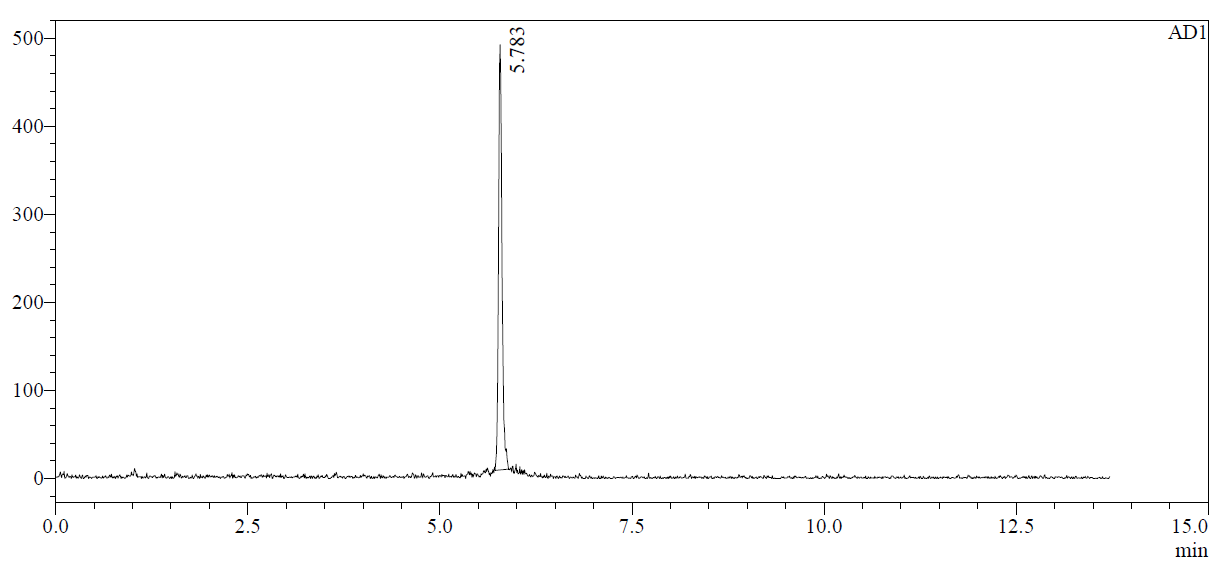


Figure 11. Radio-HPLC trace of [^68^Ga]Ga-JB-1498 2.5 hours after synthesis demonstrating no significant radiolysis compared to Figure 10.
